# Evaluation of a Voltametric E-Tongue Combined with Data Preprocessing for Fast and Effective Machine Learning-Based Classification of Tomato Purées by Cultivar

**DOI:** 10.3390/s24113586

**Published:** 2024-06-02

**Authors:** Giulia Magnani, Chiara Giliberti, Davide Errico, Mattia Stighezza, Simone Fortunati, Monica Mattarozzi, Andrea Boni, Valentina Bianchi, Marco Giannetto, Ilaria De Munari, Stefano Cagnoni, Maria Careri

**Affiliations:** 1 Department of Engineering and Architecture, University of Parma, 43124 Parma, Italy; giulia.magnani1@unipr.it (G.M.); mattia.stighezza@unipr.it (M.S.); andrea.boni@unipr.it (A.B.); valentina.bianchi@unipr.it (V.B.); ilaria.demunari@unipr.it (I.D.M.); 2 Department of Chemistry, Life Sciences and Environmental Sustainability, University of Parma, 43124 Parma, Italy; chiara.giliberti@unipr.it (C.G.); davide.errico@unipr.it (D.E.); simone.fortunati@unipr.it (S.F.); monica.mattarozzi@unipr.it (M.M.); maria.careri@unipr.it (M.C.)

**Keywords:** electronic tongue, voltametric sensors, classification, cultivars, gold nanoparticles, copper nanoparticles, multivariate chemometric tools, machine learning, linear discriminant analysis

## Abstract

The potential of a voltametric E-tongue coupled with a custom data pre-processing stage to improve the performance of machine learning techniques for rapid discrimination of tomato purées between cultivars of different economic value has been investigated. To this aim, a sensor array with screen-printed carbon electrodes modified with gold nanoparticles (GNP), copper nanoparticles (CNP) and bulk gold subsequently modified with poly(3,4-ethylenedioxythiophene) (PEDOT), was developed to acquire data to be transformed by a custom pre-processing pipeline and then processed by a set of commonly used classifiers. The GNP and CNP-modified electrodes, selected based on their sensitivity to soluble monosaccharides, demonstrated good ability in discriminating samples of different cultivars. Among the different data analysis methods tested, Linear Discriminant Analysis (LDA) proved to be particularly suitable, obtaining an average F1 score of 99.26%. The pre-processing stage was beneficial in reducing the number of input features, decreasing the computational cost, i.e., the number of computing operations to be performed, of the entire method and aiding future cost-efficient hardware implementation. These findings proved that coupling the multi-sensing platform featuring properly modified sensors with the custom pre-processing method developed and LDA provided an optimal tradeoff between analytical problem solving and reliable chemical information, as well as accuracy and computational complexity. These results can be preliminary to the design of hardware solutions that could be embedded into low-cost portable devices.

## 1. Introduction

In recent years, the electronic tongue (E-tongue) has emerged as an innovative multisensory device that can mimic human taste perception [1,2,3], realizing the objectification of sensory evaluation. This property makes food quality analysis one of the most frequent and primary applications of E-tongue, aimed at automatic qualitative and quantitative analysis of complex liquid samples and recognition of their properties [3,4,5,6].

According to the International Union of Pure and Applied Chemistry (IUPAC) definition, an E-tongue is a device combining a number of low-selective sensors and advanced mathematical signal processing procedures based on pattern recognition and/or multivariate data analysis methods such as principal component analysis (PCA) or artificial neural networks (ANNs) [7]. IUPAC’s definition fostered the development and application of the E-tongue as a modern instrument for evaluating the quality and authenticity of food product. An objective evaluation of food quality has always been of utmost importance to ensure the safety of food consumption, especially in contexts such as those of food industry, particularly for routine quality control of products [8,9,10]. Within this picture, the need for analytical tools for reliable, fast, and low-cost analysis of multicomponent samples has emerged. Portable E-tongue devices have been deemed particularly suitable for this purpose because of their ability to acquire measurement data without requiring installation in specialized laboratories [8]. An E-tongue does not give information on sample composition but rather provides a digital fingerprint of a sample, which can be used for discrimination or classification purposes and to comprehensively evaluate the shelf life of food [3].

As for the sensor array that generates multidimensional information, a wide variety of chemical sensor types is used for the design of electronic tongue systems [1,2,3,4,6,7,8,11]. Electrochemical sensors, especially potentiometric and voltametric ones, still remain the most widely used to design E-tongues [4,5,6,11,12]. Voltametric sensors are advantageous for multi-target analysis because of their high selectivity and low detection limits, various modes of measurement, and the possibility of modifying the surface of the electrodes with various chemosensitive materials, obtaining sensors of different sensitivity and selectivity towards a variety of redox-active species [4,5,6,11,13]. Over time, other chemical sensors, i.e., optical, piezoelectric, and those based on electrochemical impedance spectroscopy, have also been exploited [14]. Finally, the potential of multi-transduction electronic tongue systems, the so-called hybrid E-tongues, has been investigated for different analytical tasks [15,16]. In addition, as recently reviewed, the concept of bioelectronic tongues has attracted increasing scientific attention for food control and characterization [17]. Bioelectronic tongues include one or more biosensors in the sensor array: the embedded biorecognition components involving enzymes, antibodies, or artificial biomimetic receptor layers respond to an individual or a group of closely related analytes, thus providing valuable information together with chemometric tools.

Concerning the analysis of the signals generated by sensor arrays, since E-tongue measurements provide multidimensional data, the multivariate approach offered by chemometrics is needed [18]. Processing data produced by the sensor array follows a workflow consisting of different steps: i. extraction of salient information from the raw input data (feature extraction), ii. data pre-treatment, and, finally, iii. data assignment to one in a finite set of classes (classification) or estimation of the value of a specific quality index or physical property, e.g., concentration, based on the values of such features (regression) [19,20,21,22,23,24,25,26,27,28,29,30,31,32,33,34]. Each step should be carried out with caution due to possible errors that strongly influence measurement results.

Multivariate statistical techniques have been investigated for the quantification of some taste-related properties, such as sweetness [19], and to test the ability of the electronic tongue and attenuated total reflectance–Fourier transform infrared spectroscopy (ATR–FTIR) both to classify new tomato cultivars based on their sugar and acid profile and to predict the chemical composition of tomato samples [20]. Among chemometric tools in E-tongue data analysis, Principal Component Analysis (PCA) [21] is the most common for feature extraction. In contrast, Machine Learning methods, with frequent use of Support Vector Machines (SVM) [22], represent the prevalent choice for classification and/or regression. Among the classification techniques, ANNs and statistical methods that perform prior data analysis are also common. Research studies on orange beverages and vinegar [23], honey [24], Tieguanyin tea [25], mutton and pork meats [26], fruit juice [27], and umami taste [28] are some examples. Regarding other types of machine learning (ML) classification algorithms, the most common choice in literature appears to be SVM, often outperforming other methods due to its effectiveness in high dimensional spaces, which is a common characteristic of datasets built on E-tongue signals. Some works which follow this approach deal with the application of E-tongues to cherry tomato juices [29] and to beer classification [30]. Regression methods, such as partial least square regression (PLSR), are also sometimes used to predict the properties of food products by the response obtained by E-tongue, as shown in some works regarding white wines [15] and yogurt [31]. Finally, linear discriminant analysis (LDA) is a supervised classification algorithm commonly used to perform dimensionality reduction, enabling easier subsequent classification [32]. Research projects focusing on cattle meat [33] and tomato [34] are good examples of the application of this approach.

In the context of the research activities undertaken within the “Strengthening of the Italian Research Infrastructure for Metrology and Open Access Data in Support of Agrifood” project (METROFOOD-IT), the present work aimed to investigate the potential of a voltametric E-tongue associated with a custom data preprocessing stage to rapidly discriminate tomato purées between cultivars of different economic value. The E-tongue was implemented on a 3-channel multi-sensing platform of screen-printed carbon electrodes (SPCE) modified with gold nanoparticles (GNP) [35], copper nanoparticles (CNP) [36] and bulk gold subsequently modified with poly(3,4-ethylenedioxythiophene) (PEDOT) [37,38] conducting polymer, considering the electroactive substances present in tomato, namely ascorbic acid among water-soluble antioxidants and glucose and fructose among soluble monosaccharides [39]. Multivariate and artificial intelligence techniques were applied to the voltametric dataset to develop and validate classification models for classification of commercially available tomato cultivars according to their sugar and ascorbic acid profile. LDA performed better than the other methods, closely followed by random forest (RF) and SVM. The different sensors, when considered independently, showed different performances in tomato variety classification.

Finally, the information provided by the electronic tongue was compared with that of the ion chromatography with pulsed amperometric detection (IC-PAD) reference technique to investigate the reliability of the sample classification model.

## 2. Materials and Methods

### 2.1. Materials

Sodium chloride (NaCl), disodium hydrogen phosphate (Na_2_HPO_4_), hydrochloric acid (HCl, 37%), sodium hydroxide (NaOH), lithium perchlorate (LiClO_4_), 3,4-Ethylenedioxythiophene (EDOT), potassium chloride (KCl), potassium dihydrogen phosphate (KH_2_PO_4_), copper (II) sulfate (CuSO_4_) chloroauric acid (HAuCl_4_), ascorbic acid, D(+)glucose, D(−)fructose and NaOH 50% *w*/*w* solution in H_2_O were purchased from Sigma-Aldrich (Milan, Italy). Sulfuric acid (H_2_SO_4_) was purchased from Carlo Erba (Milan, Italy).

Phosphate Buffered Saline (PBS) was prepared with the following composition: 137 mM NaCl; 2.7 mM KCl; 1.2 mM KH_2_PO_4_; 8 mM Na_2_HPO_4_ (pH adjusted to 7.4 with HCl).

Ultrapure water (18.2 MΩ cm) used in all experiments was obtained from a Direct Q^®^ 3 UV ultrapure water system.

Phenex-RC syringe filters (26 mm diameter, 0.2 µm) were purchased from Phenomenex (Castel Maggiore, Bologna, Italy).

Screen-printed Carbon Electrodes DRP-C110 (SPCE DRP-C110) were purchased from Metrohm Italiana Srl (Origgio, Varese, Italy).

### 2.2. Sample Preparation

Three tomato (*Solanum lycopersicum*) cultivars were considered, namely ‘perino tomato’, ‘red datterino tomato’, and ‘yellow datterino tomato’, for the homemade preparation of their respective purées.

A food processor (Thermomix, TM5, Vorwerk, Wuppertal, Germany) was used to prepare the purées. In detail, 1000 g of fresh tomatoes of each variety were washed, added with 20 g of NaCl, and poured into the bowl of the food processor. Cooking was performed at 100 °C for 35 min, with mixing speed 1. At the end of cooking, the mixture was blended at speed 9 for one minute, and the purées were transferred while still hot into 250 g glass jars with caps screw, sealed, and stored until analysis.

Several 30 g aliquots of the samples were transferred to Falcon 50 mL conical centrifuge tubes and centrifuged with 5810R Eppendorf Centrifuge, purchased from Eppendorf (Hamburg, Germany). The supernatant was collected and stored at −20 °C.

The samples were diluted 1:1 in 1 M NaOH (pH = 13) to perform the analysis based on the use of GNP- and CNP-modified SPCEs and diluted 1:1 in PBS (pH = 7.4) in the case of PEDOT-modified SPCEs.

Prior to ICP-PAD analysis, sample pre-treatment consisted of centrifugation, filtration of the supernatant using 0.20 µm Phenex-RC filters and 100,000-fold dilution in ultrapure MilliQ water. Calibration standards of glucose and fructose were processed using the same 0.20 µm Phenex-RC filters.

### 2.3. E-Tongue Multi-Sensor Platform

The developed multi-sensor platform includes three different sensors implemented on commercial SPCEs electrochemically modified with (i) GNP, (ii) CNP, and (iii) bulk gold subsequently coated with electropolymerized poly(3,4-ethylene dioxythiophene) (PEDOT).

The modification of SPCEs and the voltametric readout were performed using a Multi Autolab M204 multichannel potentiostat/galvanostat (Metrohm Italiana Srl, Origgio, Varese, Italy) using NOVA 2.1.6 Advanced Electrochemical Software. The SPCEs were connected to a connector box from DropSens, Origgio, Varese, Italy (DRP-DSC), allowing their interfacing with the potentiostat.

As for GNP-modified SPCEs, gold nanoparticles were electrodeposited onto the electrode surface by drop casting 50 µL of 5 mM HAuCl4 in 100 mM KNO_3_ and scanning the potential by cyclic voltammetry (CV) ranging from −0.7 to +0.4 V for 10 scans with a scan rate of 50 mV/s. GNPs were then activated with a solution of 0.1 M H_2_SO_4_ by CV (+0.1–+1.5 V, 5 scans, scan rate: 100 mV/s)

CNP-modified SPCEs were obtained by drop casting 50 µL of 10 mm CuSO_4_ in 0.1M H_2_SO_4_ onto the SPCEs through galvanostatic electrodeposition (−225 µA, duration: 60 s, interval time: 0.1 s.). Activation of CNPs was performed in 0.1 M NaOH by CV (0–+1 V, 20 scans, scan rate: 100 mV/s).

PEDOT electrodeposition required two steps. First, bulk gold was deposited onto the surface of SPCEs by chronoamperometry (−1.6 V; duration: 200 s; interval time: 0.1 s) by drop casting 50 µL of 5 mM HAuCl_4_ in 100 mM KNO_3_ solution. The gold surface was then activated by dipping the electrodes in 1.2 M NaOH and subsequently in 1.2 M HCl for 5 min each. Then, electropolymerization of EDOT was performed by CV drop casting 50 µL of a 5 mM EDOT solution in 0.1 M LiClO_4_ and scanning the potential between −0.5 and +1.25 V with a scan rate of 100 mV/s for 10 scans. After each step, electrodes were rinsed with distilled water and dried with a nitrogen stream.

### 2.4. Raw Data Acquisition

All tomato pureés were analyzed by cyclic voltammetry using the three different sensors of the multi-sensing platform. To this purpose, 50 µL of each diluted sample were drop-casted onto the SPCEs modified with GNP, CNP, and bulk gold-PEDOT.

As for GNP- modified SPCEs, aliquots of independent samples were tested by cyclic voltammetry from −0.5 to +0.6 V, whereas the scans ranged from 0 to 1 V for CNP-modified SPCEs. In both cases, a scan rate of 50 mV/s was used.

As for PEDOT-modified SPCEs, cyclic voltammograms were recorded by applying a reduction potential of −0.5 V for 30 s before acquiring scans from −0.5 to +0.5 V at a scan rate of 50 mV/s. A total of 1080 CV scans (120 × 3 × 3) were acquired considering the acquisition of 120 scans from each of the three sensors for each type of tomato purée. In particular, after acquiring 20 scans, each modified SPCE was discarded and replaced with an analogous sensor. To this aim, six different aliquots of the same stock of purée were analyzed for each cultivar, with as many independent sensors. Measurements were performed in randomized order. Concerning the time involved in the acquisition of the voltametric data, each scan required 20 s resulting in a total of 40 s per complete CV cycle.

### 2.5. Analysis of Monosaccharides by IC-PAD

Soluble monosaccharides (glucose and fructose) in tomato purée samples were determined by IC-PAD using a DIONEX/ICS-6000 chromatograph (Thermo Fisher Scientific, Waltham, MA, USA) equipped with an electrochemical detector, a column heater, a standard injection six-port valve for analytical IC systems with a 25 μL sampling loop, an anion-exchange Dionex CarboPac™ PA1 column (4 × 250 mm) and a Dionex CarboPac™ PA1 guard column (4 × 50 mm).

The mobile phase was prepared by adding 50% *w*/*w* NaOH solution to 18.2 MΩ water degassed with nitrogen, to yield a final concentration of 200 mM NaOH. Chromatographic separation was performed under isocratic conditions using 200 mM aqueous NaOH at a flow-rate of 1 mL/min. The run time was 10 min per sample. Column was thermostated at 30 °C. Injection volume was 25 µL.

The electrochemical detector was equipped with a gold working electrode, an Ag/AgCl reference electrode and a titanium cell body working as counter-electrode. The cell compartment was thermostatted at 20 °C. A quadruple waveform was chosen with the following time and voltage parameters: 0.10 V from 0 to 40 s, −2.00 V from 0.41 to 0.42 s, +0.60 V at 43 s and −0.10 V from 0.44 to 0.50 s, with current integration from 0.20 s to 0.40 s.

Calibration standards of glucose and fructose, prepared in ultrapure MilliQ, as well as tomato purée samples, were analyzed in triplicate and the monosaccharide concentration was expressed as g/100 g. All standards and samples were subjected to the procedure as described under Sample Preparation (Section 2.2). Linear ranges of 0.05–1 mg/L were obtained for glucose and fructose.

Chromeleon 7.2.10 software (Thermo Fisher Scientific, Waltham, MA, USA) was used for system control and data processing.

The results of the reference technique were analyzed using analysis of variance (ANOVA).

### 2.6. Machine Learning Algorithms and Raw Data Preprocessing

Several data classification methods were compared to derive a model that could effectively recognize the cultivar of tomato purée samples in the dataset.

The RF classifier [40] derives a set of independent decision tree (DT) classifiers [41] that use Gini’s criterion to build a classification ensemble model [42] from the training data.

The SVM algorithm [21] is a supervised learning method that performs a linear domain bisection of the dataset based on the equation $y=wx^{\prime }+\gamma$ where x are the input data, γ a penalization coefficient based on the classification results obtained, and w are the model parameters.

A recurrent neural network (RNN) [43] is a particular type of NN in which the neurons’ output signals can also be fed back as additional input to the same or previous nodes, instead of being fed only to the neurons topologically closest to the output as in the Feedforward Networks or Multilayer Perceptrons.

PCA is a statistical technique widely used in chemometrics and many other fields [44] to reduce data dimensionality. It works by applying a linear transformation from the original data space to a new space represented by the eigenvectors of the covariance matrix of the data.

LDA [32] is a feature reduction and classification method based on a linear data transformation like PCA. However, the main difference with PCA is that, while PCA is unsupervised and aimed at an efficient data representation, LDA is supervised, i.e., it considers the data/classes associations to build a model that explicitly maximizes the inter-class distance and thus data “classifiability”.

In all experiments, we used the Python implementations of the algorithms offered by the Scikit-learn library. Tests were run on a computer with an Intel i7 CPU running at 3.2 GHz and 32 GB RAM.

**Figure 1:**
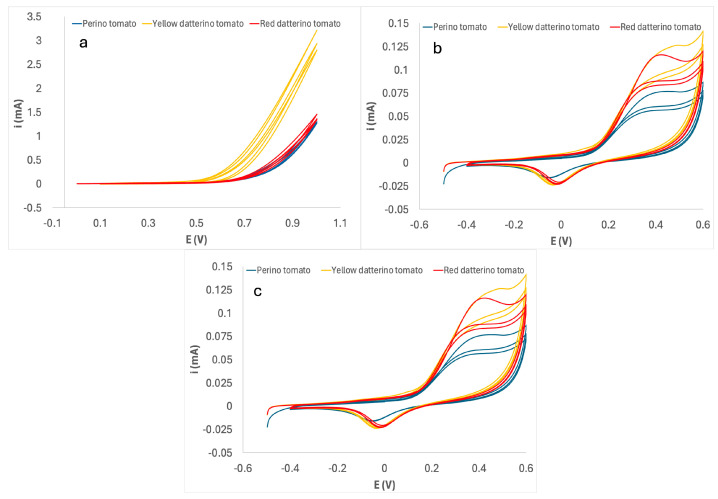
Cyclic voltammetry of the different tomato purée samples using (**a**) CNP, (**b**) GNP, and (**c**) PEDOT-modified SPCEs.

The application of the ML algorithms to the raw data acquired by the E-tongue device, shown in Figure 1, would not be an optimal choice from two viewpoints. On the one hand, the typical redundancy of raw data and the noise that affects them intensify the problems posed by the variability that characterizes data describing any natural phenomenon. On the other hand, aiming at a possible future implementation on a low-cost hardware platform, the computational complexity of the algorithm should be reduced as much as possible. A preprocessing stage including feature reduction can reduce the computational complexity [45] of the algorithm without affecting performance or even improving it. Therefore, the acquired data had to be appropriately processed before undergoing classification.

**Figure 2:**
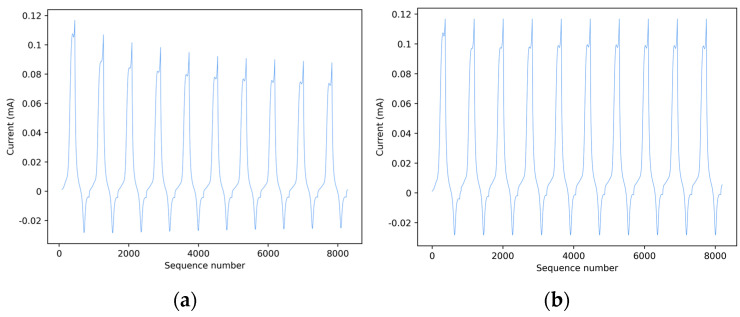
Current vs. sequence number representation of the measurements of a perino tomato purée acquired on GNP-modified SPCE before (**a**) and after (**b**) normalization to the range of the first scan.

An obvious observation is that in the current vs. potential representation of a scanning cycle, each measurement cannot be considered a sample of a function I = f(V) since the values of current I corresponding to a given potential V are different when measured during the forward and backward scans, evidencing a hysteresis-like behavior. This could represent a problem for ML algorithms, which should treat the input as a two-dimensional data set. Models developed to explicitly deal with spatial representations are not appropriate for this case since the measurements, consisting of signals from single electrodes, are inherently monodimensional. For these reasons, measurement time series representing current vs. potential relationships were transformed into relationships between current vs. sequence number, as shown in Figure 2.

Figure 2a shows data from a complete voltametric cycle, displayed as current vs. sequence number, the latter representing the index of a data point within the vector that contains the acquired data. Each voltametric cycle comprises a series of scans according to the aforementioned relationship, in which the amplitude of the measured currents decays significantly with time, mainly due to diffusion phenomena and, to a lesser extent, to the progressive depletion of the sensing layer. Observing the decay of the measured peak currents in subsequent measurements it appears that if raw data were used directly for training, a complete measurement consisting of a sequence of scans would represent a single element of the dataset (i.e., in machine learning jargon, a training instance). Instead, the pattern of the curve representing a scan can be considered invariant with respect to the scan number, which means that every single scan contains the same information regarding the chemical composition of the analyzed sample. Consequently, if the scans are rescaled within the same range as the first, it is possible to consider each scan as an instance of the same process. As a result, this preprocessing stage increases the number of training instances available to the ML algorithm by one order of magnitude, offering the additional/alternative option of predicting the cultivar from each scan individually and then selecting the most frequently predicted class as the result of the classification for the whole measurement. Figure 2b shows the effect of this normalization process.

**Figure 3:**
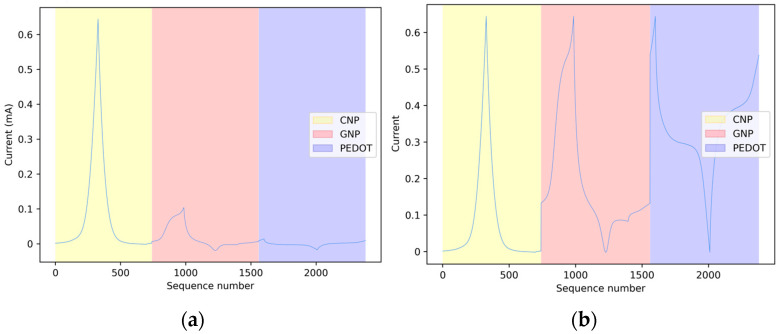
Concatenation of single scans from the three sensors in a current vs. sequence number representation before (**a**) and after (**b**) normalization to the range of the CNP-modified sensor type.

Furthermore, to collect the full range of information on a measurement, single CV scans from each of the three sensor types, as those represented by the peaks in Figure 2, were concatenated to create a unified data set. Each sample of this data set is the result of the concatenation of three scans acquired by the CNP-, GNP-, and PEDOT-modified SPCEs, respectively, as shown in Figure 3a.

It should be noted, however, that this procedure may potentially give rise to an issue due to the differing current ranges of the scans originating from the other sensors. Indeed, in several ML algorithms, using data represented over different ranges can result in a bias in the influence of data originating from the highest-amplitude source. As shown in Figure 3a, the range of the current values for the GNP and PEDOT electrodes is considerably smaller than that of the CNP-modified SPCEs; this gap can cause a classifier to assign different weights to the contributions of the three sensors, negatively affecting the classification outcomes. Hence, to compensate for this, the three sequences were also normalized by equalizing the range of measurements from the GNP- and PEDOT- modified SPCEs to the range of the measurements from CNP-modified SPCEs, as shown in Figure 3b.

Finally, since a final preprocessing stage limits the computational complexity of the method by reducing the number of features to be fed into the ML algorithm, the input sequences were subsampled by averaging the sample values over subsequent windows, the length of which was optimized during the training phase. This means that, if the optimal subsampling window is found to be of length *k*, the final length of each sample is reduced to ⌊*n/k*⌋ data points. In this case, the best *k* was found to be 35 points.

The ML techniques described in Section 2.5 were finally applied to the preprocessed data.

## 3. Results and Discussion

### 3.1. Features of the Multi-Sensing Platform

The E-tongue was developed using low-cost SPCEs as electrodic substrates to maximize the performance/cost ratio of the multi-sensor platform. To evaluate the potential of the electronic tongue in discriminating tomato purées of three different cultivars in terms of value and cost, the characteristics of the gold and copper nanoparticles and of PEDOT as a conductive polymer were exploited to study the voltametric profile of the tomato products on the electrodes [46]. For this purpose, the electroactive substances present in tomatoes were considered, namely ascorbic acid among water-soluble antioxidants and glucose and fructose among soluble monosaccharides.

Specifically, gold and copper nanoparticles play a peculiar electrocatalytic activity toward the non-enzymatic oxidation of soluble monosaccharides, mainly represented by glucose and fructose in tomato fruits. The oxidation mechanism provides the dehydrogenation of the hemiacetal group on the anomeric carbon of the sugar and involves CuO generated by the oxidation of CNPs, which is converted into Cu(III) species. Such species, in turn, act as redox mediators for the oxidation of glucose into gluconolactone, for example [36].

Similar mechanisms involve gold-mixed oxides *Au_n_O_m_* generated by the oxidation of GNPs as oxidized species active in redox mediation for monosaccharide oxidation. Classically, electrocatalysis is reported to be mediated by a layer of hydrated oxide formed by oxidizing the surface of the electrode, on which glucose is adsorbed [47]. The electrocatalytic activity at CNP and GNP electrodes is favored in a highly alkaline medium, fundamental for generating the reactive oxides on the surface and for neutralizing the protons formed during monosaccharide dehydrogenation steps [48].

Other electroactive organic molecules that characterize tomato samples and guarantee the maintenance of their sensorial quality are antioxidants, mainly represented by ascorbic acid (AA), which is considered the most important water-soluble antioxidant contained in fresh tomatoes. The AA content of fresh fruits depends on the variety and the cultivation conditions [49]. It decreases during thermal processing, temperature and oxygen being the main factors responsible for ascorbic acid losses [50].

In this work, PEDOT-modified SPCEs, which constitute the third sensor of the multi-sensor platform, were tested to investigate their contribution to the discrimination between the tomato purées of different cultivars because of the electrocatalytic activity of PEDOT-modified electrodes towards the oxidation of ascorbic acid [51]. SPCEs were functionalized with a composite material obtained by combining an electrodeposited gold layer and electropolymerized 3,4-ethylenedioxythiophene (EDOT) on the modified surface, where the metal layer acts as a substrate for the electropolymerization of EDOT. The composite polymer thus obtained (Au-PEDOT) is known to exhibit excellent ability towards the electrocatalytic oxidation of AA, as the gold layer acts as an enhancer to trigger an electrocatalytic cycle in which PEDOT acts as a redox mediator for the electrochemical oxidation of AA.

The significant electrocatalytic effect ascribable to PEDOT is attested by an earlier oxidative response of AA and by a noticeable increase in the anodic peak current compared to bare SPCEs. Furthermore, the electrocatalytic properties of PEDOT allows to overcome the drawbacks related to AA determination on bare electrodes due to the fouling of the electrode by oxidation products and the high potential required for AA oxidation, which can cause interferences, leading to poor selectivity in complex matrices such as food samples [37,51].

### 3.2. Application of ML Algorithms to the Preprocessed Data: Classification of Tomato Purees as a Case Study

The raw voltametric data were acquired using the sensors described in Section 3.1. Data were then preprocessed and reorganized into a dataset suitable for tomato classification purposes.

The preprocessing applied to the data set comprises several steps. Each raw measurement is composed of several voltametric responses of the three sensors. The first preprocessing step consists of separating the single voltametric scans, linearly normalizing each subsequent scan to the range of the first one according to the following equation:

(1) $$s_{i}^{N}=\frac{\left(max(f)-min(f)\right)(s_{i}-min(s))\;}{\max\left(s\right)-min(s)}+min(f)$$

where *s* is the scan to be normalized, *f* is the first scan in the measurement and $s^{N}$ is the normalized scan. The scans are then associated in groups of three, one scan for each sensor (CNP-, GNP- and PEDOT- modified SPCEs, respectively). A further normalization is then performed in order to align the range of the latter two scans to the range of the first scan in the sequence, while simultaneously maintaining the information concerning the range of the aforementioned scans. Here, the normalization is performed as follows:

(2) $$s_{i,\;GNP}^{N}=\frac{\left(max\left(\mathrm{CNP}\right)-min\left(\mathrm{CNP}\right)\right)(s_{i,GNP}-min\left(\mathrm{GNP}\right))}{\max\left(GNP\right)-min(GNP)}+min(CNP)$$

(3) $$s_{i,\;PEDOT}^{N}=\frac{\left(max(CNP)-min(CNP)\right)(s_{i,\;PEDOT}-min(PEDOT))\;}{\max\left(PEDOT\right)-min(PEDOT)}+min(CNP)$$

where Equations (2) and (3) are used to normalize scans acquired with GNP- and PEDOT-modified SPCEs, respectively, *s* is the scan to be normalized, $s^{N}$ is the normalized scan, *max*(CNP) and *min*(CNP) are the maximum and minimum values of the whole training set of scans acquired with CNP-modified SPCEs. The same holds for *max*(GNP), *min*(GNP), *max*(PEDOT) and *min*(PEDOT).

The final preprocessing step consists of applying an averaging filter over moving windows to each scan and then concatenating the groups of three scans.

Regarding the classification task, several comprehensive evaluations were performed by comparing the performance of the ML algorithms described in Section 2. The metrics used for the assessment, namely Recall, Precision, and F1 score, are defined as follows:

(4) $$Recall=\frac{TP}{TP+FN}$$

(5) $$Precision=\frac{TP}{TP+FP}$$

(6) $$F1\;score=\frac{2}{{Recall}^{-1}+{Precision}^{-1}}=\frac{2TP}{2TP+FP+FN}$$

where *TP*, *FP*, and *FN* are True Positives, False Positives, and False Negatives, respectively.

Each ML method was optimized to find the best parameter settings that yield the highest F1 in the classification process. From this viewpoint, it is worth noting that ML algorithms are very general approaches whose behavior may change dramatically depending on a large number of parameters that regulate their operation.

For all tested settings, the dataset was split into a training set and a test set, with the latter comprising 30% of the samples and the training set consisting of the remaining dataset. Furthermore, to minimize the chance that intrinsic randomness of the methods could notably affect the evaluation and to ensure that the results were not biased by a specific split of the data into a training and a test set, 100 independent tests were performed for each ML method, each characterized by a different random subdivision of samples into a training set and a test set, keeping the same size ratio of the two sets. Finally, the results of all 100 runs were averaged to compute an unbiased estimate of each setting’s performance.

Table 1 compares the results obtained by applying ML techniques to raw data and preprocessed data with the custom-designed method. As can be observed, the preprocessing stage improves the metrics in almost all cases.

As regards the RF classifier, the best results were obtained using 100 estimators together with the Gini criterion to assess the quality of the data splits. After observing that limiting the depth of the estimators not only does not prevent overfitting but even worsens the classification results, no limit was imposed on the depth of the estimators.

Since the feature space is high-dimensional, an SVM was also tested using a polynomial kernel of degree 6. This choice was suggested by the complexity of the data, which makes finding a linear separation between the different classes highly unlikely.

Subsequently, an RNN was tested. In this case, the training set was further split to add a validation set used to fine-tune the parameters of the network. The same 30% ratio applied to the training-test split was used to extract a validation set from the training set, i.e., 30% of the training set was considered as the validation set. A dropout, with a rate of 0.6, was also added to prevent overfitting by randomly “disabling” some connections with each weight update, thus forcing the network to be flexible enough to compensate for the missing connections. Finally, a Softmax layer was used to generate the final network output.

**Figure 4:**
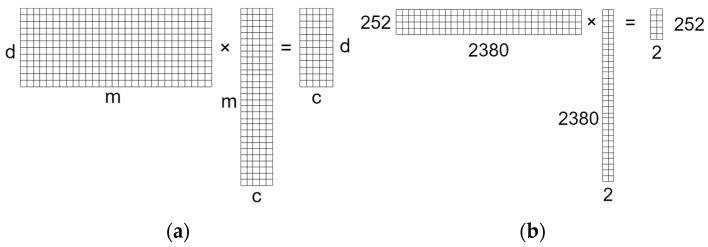
Graphical representation of the multiplication between the dataset matrix and the transformation matrix and the resulting transformed matrix. (**a**) Representation of the general case corresponding to Equation (4); (**b**) Representation of the specific case at hand using a two-component transformation matrix.

PCA was evaluated to extract features and classify data jointly with Kmeans clustering [52] with a “cluster-to-classes” approach. As shown in Figure 4, if n is the smaller value between the dimension of the data domain *m* and the number of data instances *d*, the method generates a transformation matrix $T_{m\times c}$ with c (1≤c≤n) components as a column subset of the matrix having as columns the eigenvectors of the covariance matrix sorted according to the corresponding eigenvalues’ magnitude. This means that the whole dataset is transformed according to the following matrix multiplication:

(7) $$D_{d\times m}T_{m\times c}=N_{d\times c}$$

where D is the original dataset matrix, T is the transformation matrix, and N is the resulting transformed dataset matrix. As an example, considering the training set at hand, if c=2, m=2380, d=252, each sample $S_{1\times 2380}$ in the dataset is transformed into ${S\prime }_{1\times 2}$. Thus, the two components of the transformation of each of the *m* samples in the dataset can be seen as their coordinates on the plane representing the transformed space.

Ideally, assuming that the original dataset contains enough information to separate the data, the PCA should provide a transformation that represents the original data with fewer components, reducing the dimension of the dataset while still retaining enough information for the classification to be successful. If the dimensional reduction mainly affected the noise, the classification performance could even improve. The Kmeans clustering algorithm is used to identify clusters in the transformed training set. These clusters are then assigned a label corresponding to the class to which the majority of the points assigned to the cluster belong. In the testing phase, the transformed data are assigned to one of the previously identified clusters according to a distance criterion, inheriting its label. The accuracy and F1 score are computed by analyzing the correct or erroneous class assignments.

Finally, the LDA algorithm was evaluated as a methodology similar to PCA, taking into account that LDA generates a transformation matrix maximizing inter-class separation. As with PCA, the transformed data were finally classified using the KMeans algorithm, clearly outperforming PCA. This result can be explained considering that PCA is an unsupervised method that produces a data transformation resulting in a reduced-size representation that optimally preserves information content, while not being necessarily optimal for classification. In contrast, the transformation produced by LDA in a supervised way maximizes the inter-class distance, which is strongly correlated with classification accuracy.

**Figure 5:**
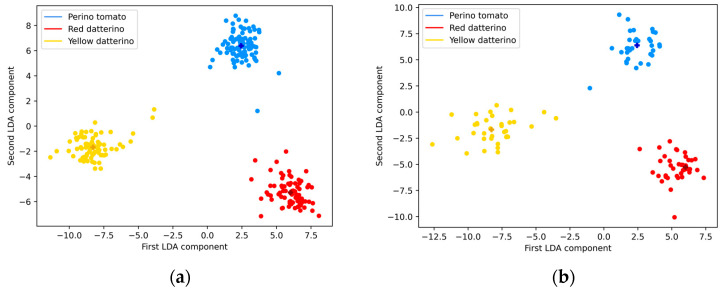
Two-component LDA representation of the training set (**a**) and test set (**b**) relevant to tomato purée classification by cultivar.

Figure 5a displays the score plot for the training dataset processed by the LDA algorithm by which two components could be computed since we are discriminating three classes. The centroids found by the KMeans algorithm are represented by the darker cross-shaped points. The plot clearly shows a separation between the three different classes. The two LDA components explain, respectively, 58.4% and 41.6% of the total variance. The test data is also shown in Figure 5b and exhibits the same clear separation. In this step, the test data was transformed using the model generated from the training set and classified based on the Euclidean distance to the cluster centroids calculated from the training data.

It should be noted that LDA allows the analysis of the transformation matrix and the direct evaluation of the features contributing more efficiently to tomato pureé classification.

**Figure 6:**
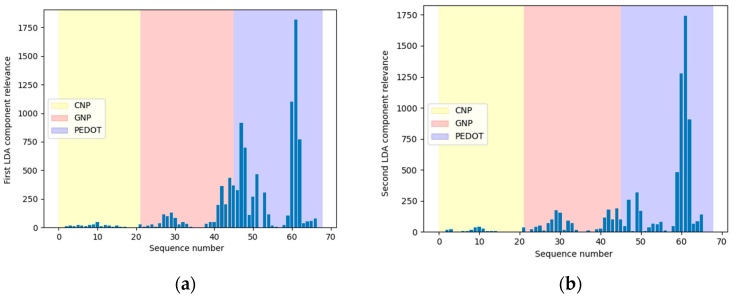
Relevance of the features of the first (**a**) and the second (**b**) component of the transformation matrix relevant to tomato purée classification by cultivar.

Figure 6a,b show the first and second components of the obtained transformation matrix using a 35-fold subsampling for visualization convenience. As can be observed, the PEDOT-modified SPCE for the detection of ascorbic acid is the sensor that produces the descriptor variables with the highest weight (coefficient) values compared to the CNP- and GNP-modified sensors for the measurement of soluble monosaccharides.

**Figure 7:**
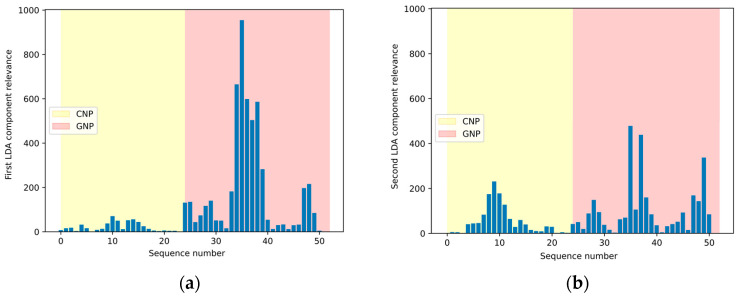
Relevance of the features of the first (**a**) and the second (**b**) component of the transformation matrix relevant to tomato purée classification by cultivar after excluding data from the PEDOT sensor.

In order to test the robustness of the classification model and verify its possible overreliance, the results obtained by excluding the data component with the highest relative weight on classification (i.e., data acquired from the PEDOT-modified SPCE) were analyzed. The performance of a new classification model based only on the outputs of the sensors modified with CNP and GNP was then evaluated in relation to the monosaccharides content of the samples under study. Figure 7a,b demonstrate the impact of removing the data generated by the PEDOT electrode on the transformation matrix. The GNP electrode now has a more significant impact on classification. It is worth noting that the average F1 score computed over 100 independent executions of the classifier decreases only slightly to 98.27% with respect to using all three data components.

Besides outperforming other methods, LDA is also preferable because the resulting model only consists of the transformation matrix and the cluster centroid coordinates, making classification of new samples straightforward, with significant benefits for the cost-efficiency of hardware implementation on a microcontroller as part of the firmware of a portable device, which is a possible future extension of this work. With such a model available, the samples can be transformed by computing the scalar product between the sample and the transformation matrix. A generic sample can then be labelled based on its distance from the cluster centroids by assigning it the label associated with the closest cluster.

In contrast, the RF Classifier, which also yields a good F1 score, relies on a large number of individual DT Classifiers, specifically 100 in our case, and has no limit on the maximum depth. Therefore, implementing RF classifiers in hardware could be more complex and computationally expensive than LDA.

A comparison with the literature was carried out to highlight the effectiveness of the proposed preprocessing method. Recent works that exploit E-tongue and LDA to detect food adulteration or for food classification were selected. For instance, a study on honey adulteration detection was recently described where LDA was applied, achieving an accuracy of 96.55% [53]. In another interesting piece of research dealing with meat classification from five cattle breeds, LDA was able to classify the different meat origins with a 97.52% average prediction accuracy [33]. Finally, LDA achieved a discrimination accuracy of 91.67% in a study aimed at discriminating between soybean paste samples of various brands [19].

Notably, in these works, the performance of the methods is expressed in terms of accuracy rather than in terms of the F1 score, where accuracy is defined as follows:

(8) $$Accuracy=\frac{TP+TN}{TP+FP+TN+FN}$$

For the sake of comparison, we calculated the average accuracy of our method over 100 independent runs, in each of which the training, validation, and test sets were randomly selected. The resulting average accuracy was 99.17%, which confirmed the goodness and generality of our results.

It is worth noting that, in the evaluation of the method we developed, the data were classified by considering single scans and not whole measurements as data instances, to also demonstrate that, although signal amplitude decays over time, the information content of each single scan is the same and can be fully recovered by normalizing the scan data. This suggests that a classification strategy based on full measurements, in which the final measurement classification is obtained by a majority-vote strategy applied to the classification of single component scans, can be even more robust. This hypothesis has been confirmed by the perfect 100% accuracy obtained using such an a posteriori measurement-wise classification of our data.

### 3.3. Model Cross-Validation by IC-PAD Analysis of Soluble Monosaccharides

In order to investigate the reliability of the sample classification based on the dataset restricted to the signals obtained with the SPCEs functionalized with GNPs and CNPs, a cross-validation of the classification model was carried out by performing quantitative analysis of glucose and fructose.

To this aim, the tomato pureé samples were analyzed by IC-PAD, a well-established technique for the determination of carbohydrates, which are detected by measuring the electrical current generated at a gold working electrode in a basic medium [54,55].

Table 2 shows the results obtained from glucose and fructose determination in the pureé samples.

A one-way analysis of variance was performed on the sum of glucose and fructose concentrations, proving a significant difference (*p* < 0.01) in soluble monosaccharide content among tomato pureés of different cultivars. It is interesting to observe how the data obtained confirm that the differences in the monosaccharide content of tomato pureés of different cultivars justify the robustness of a classification model based on datasets deriving from sensors whose response is based on electrocatalytic processes involving sugars present in the samples.

## 4. Conclusions

A voltametric E-tongue coupled with a custom data pre-processing stage to improve the performance of ML techniques was successfully applied to the classification of tomato purées of cultivars of different economic value based on soluble monosaccharide content. A first classification model indicated the PEDOT electrode for the detection of ascorbic acid as the variable with the greatest weight compared to the CNP- and GNP-modified electrodes for the detection of glucose and fructose. Further investigations have shown that the two-sensor electronic tongue implemented on commercial SPCEs electrochemically modified with GNP and CNP was able to differentiate samples of different cultivars based on soluble monosaccharides, with the GNP electrode having the most significant impact on classification. It should be noted that the average F1 score computed over 100 independent executions of the classifier decreases only slightly to 98.27% with respect to using all three data components. The developed electronic tongue has proven to be a rapid and easy-to-apply technique, without the need for laborious sample preparation and experienced operators.

Among artificial intelligence techniques and chemometric tools applied to the voltametric dataset, the results of the LDA technique mostly indicate better performance than RF and SVM.

These findings show that a custom preprocessing stage may improve the performance of ML techniques for classification purposes, paving the way for food adulteration control using an E-tongue. The case study regarding the classification of different tomato cultivars using E-tongue analysis of pureés from three different varieties demonstrated that such association can be highly effective, with an average F1 score of 99.26%, meaning that most scans were correctly classified. Furthermore, the preprocessing stage reduces the number of input features, lowering the computational cost of the entire method and fostering future cost-efficient hardware implementation.

Considering a possible extension of this study to a broader variety of tomato cultivars or even blends of cultivars, it is worth noting that the software developed for training the model was written according with reusability in mind, i.e., it was structured to work with any number of classes, independently of the specific food category to be analyzed, since it self-configures based on the labels used in the input dataset.

To even better face the important challenges related to food authenticity analysis using electrochemical sensors, future developments will include the integration of the developed E-tongue device with an E-nose device to combine the data obtained with these tools into a more complete overall sensory analysis platform.

## Figures and Tables

**Table 1:** Comparison of the results obtained by applying classification algorithms on raw and preprocessed data.

ML Algorithms	Without Preprocessing			With Preprocessing		
	F1 Score	Recall	Precision	F1 Score	Recall	Precision
LDA with KMeans	68.31%	68.33%	79.84%	99.26%	99.26%	99.28%
RF	80.53%	81.08%	82.23%	98.25%	98.28%	98.26%
SVM	33.19%	42.27%	61.07%	97.68%	97.73%	97.74%
RNN	38.78%	12.04%	72.22%	78.38%	76.85%	78.30%
PCA with KMeans	32.78%	42.46%	29.12%	20.17%	31.16%	19.96%

**Table 2:** Glucose and fructose concentrations determined in the tomato pureé samples by IC-PAD. Means and standard deviations from 3 replicated measurements.

Pureé Sample (Tomato Cultivar)	Glucose (g/100 g)	Fructose (g/100 g)
Perino	2.62 ± 0.03	2.41 ± 0.03
Red datterino	3.37 ± 0.03	4.02 ± 0.04
Yellow datterino	3.92 ± 0.02	4.17 ± 0.11

## Data Availability

Data are contained within the article.
